# Role of Kupffer cells in tolerance induction after liver transplantation

**DOI:** 10.3389/fcell.2023.1179077

**Published:** 2023-08-03

**Authors:** Weixiong Zheng, Lingxiang Yang, Shiming Jiang, Mingxiang Chen, Jinzheng Li, Zuojing Liu, Zhongjun Wu, Jianping Gong, Yong Chen

**Affiliations:** ^1^ Department of Hepatobiliary Surgery, The First Affiliated Hospital of Chongqing Medical University, Chongqing, China; ^2^ Department of Hepatobiliary Surgery, The Second Affiliated Hospital of Chongqing Medical University, Chongqing, China

**Keywords:** Kupffer, liver transplantation, immune tolerance, macrophage polarization, acute rejection

## Abstract

Currently, liver transplantation has reached a level of maturity where it is considered an effective treatment for end-stage liver disease and can significantly prolong the survival time of patients. However, acute and chronic rejection remain major obstacles to its efficacy. Although long-term use of immunosuppressants can prevent rejection, it is associated with serious side effects and significant economic burden for patients. Therefore, the investigation of induced immune tolerance holds crucial theoretical significance and socio-economic value. In fact, the establishment of immune tolerance in liver transplantation is intricately linked to the unique innate immune system of the liver. Kupffer cells, as a crucial component of this system, play a pivotal role in maintaining the delicate balance between inflammatory response and immune tolerance following liver transplantation. The important roles of different functions of Kupffer cells, such as phagocytosis, cell polarization, antigen presentation and cell membrane proteins, in the establishment of immune tolerance after transplantation is comprehensively summarized in this paper. Providing theoretical basis for further study and clinical application of Kupffer cells in liver transplantation.

## 1 Introduction

Since Professor Thomas Starry’s groundbreaking liver transplantation operation at the University of Colorado in 1963 (Bodzin and Baker, 2018), advancements in surgical methods and techniques have led to a gradual increase in the success rate of liver transplants. However, lifelong use of immunosuppressants remains necessary to prevent rejection and improve survival rates for liver transplant recipients (Thomson et al., 2020), despite the high economic burden and serious side effects they bring (Feng and Bucuvalas, 2017). Therefore, it is imperative to minimize or eliminate the use of immunosuppressive drugs and focus on enhancing tolerance mechanisms against foreign antigens.

Immune tolerance refers to the state where the recipient’s immune system does not mount an attack against the graft, even in the absence of immunosuppressive drugs, following exposure to donor antigens. The liver is an organ with immune privilege. In comparison to the heart, kidney, and other organs, it possesses a unique innate immune system consisting of liver-derived dendritic cells (DCs), Kupffer cells, sinusoidal endothelial cells (LSECs), liver-derived natural killer cells (NKs), natural killer T cells (NKTs), and other innate immune cells. These cells play a crucial role in the establishment of immune tolerance following liver transplantation through intricate interactions (Crispe, 2014; Zheng and Tian, 2019).

Kupffer cells, as the predominant macrophages in the liver and accounting for approximately 90% of all resident macrophages in the body, exhibit robust phagocytic and antigen-presenting capabilities akin to other macrophage populations. These cells play a pivotal role in immune regulation and tissue repair following damage. Studies have demonstrated that the immune response elicited by Kupffer cells is intimately associated with graft survival rate and establishment of immune tolerance following liver transplantation (Bilzer et al., 2006; Jenne and Kubes, 2013; Jiang et al., 2020).

In this review, we present a comprehensive summary of our research findings and integrate the conclusions from other relevant studies. Our aim is to elucidate the mechanisms by which Kupffer cells reduce rejection, induce immune tolerance, and maintain it after liver transplantation. This will provide a solid theoretical foundation for further research and clinical application of Kupffer cells in liver transplantation.

## 2 Activation of Kupffer cells after liver transplantation

The liver receives blood supply from both the portal vein and hepatic artery systems, with the former being its primary source of circulation. Due to the communication between the portal vein and the intestinal system, the liver is constantly exposed to a diverse array of microbial products, cellular metabolites, and toxins originating from the gut microbiota (Urata et al., 2000). The liver must maintain its tolerance to these antigens, thus the immune microenvironment of the liver itself exhibits a certain degree of immunological tolerance. After liver transplantation, there will be a cascade of microenvironmental changes, including the activation of Kupffer cells and dendritic cells, an increase in antigen-presenting cells, elevated levels of inflammatory mediators, regulatory T cell activation and upregulation of major histocompatibility complex (MHC) proteins (Mastoridis et al., 2017). The activation of Kupffer cells is typically associated with ischemia-reperfusion injury during liver transplantation. When the portal vein becomes obstructed, hepatocyte metabolism is disrupted due to inadequate oxygen and nutrient supply, leading to the accumulation of pathogen-associated molecular patterns (PAMPs) produced by exogenous pathogens and damage associated molecular patterns (DAMPs) released by endogenous necrotic cells such as lipopolysaccharide (LPS) and high mobility group protein 1 (HMGB1) in the gastrointestinal tract. Following reperfusion, these deleterious metabolic byproducts, particularly elevated levels of LPS, are transported to the liver via the portal vein and trigger activation of KCs (Yang et al., 2018; Zhang et al., 2021b). In addition, high concentrations of LPS indirectly activate Kupffer cells by triggering complement systems such as C3a and C5a (Bilzer et al., 2006; Thorgersen et al., 2019). When activated, KCs generate a plethora of reactive oxygen species (ROS), reactive oxygen free radicals, reactive nitrogen substances and pro-inflammatory factors such as TNF-α, IL-6 and IL-1. ROS, TNF-α and IL-1 also positively regulate the production of pro-inflammatory cytokines in KCs thereby amplifying the inflammatory cascade reaction leading to severe liver damage (Dhanasekaran, 2017).

## 3 The role of different functions of Kupffer cells in the establishment of immune tolerance after liver transplantation

### 3.1 The phagocytic function of Kupffer cells is involved in the formation of immune tolerance

As resident macrophages in the liver, Kupffer cells adhere to vascular endothelial cells and capture pathogens and apoptotic cells that circulate within the bloodstream (Mu et al., 2019). Kupffer cells phagocytose and digest large particles with potential immunogenicity, release phagosomes with antigen presentation function, and promote their own activation (Gordon, 2002; Greenberg and Grinstein, 2002). Failure to completely clear apoptotic cells result in the accumulation of apoptotic fragments and triggers an inflammatory cascade reaction. The timely clearance of apoptotic cells by activated Kupffer cells following liver transplantation is therefore a crucial factor in maintain immune tolerance (Li et al., 2017). Kupffer cells can secrete inhibitory factors, such as transforming growth factor-β, interleukin-10 and prostaglandin-2 (Atkin-Smith, 2021), following phagocytosis of apoptotic cells. This results in the creation of a local immunosuppressive microenvironment that reduces lymphocytes function and induces specific immune tolerance to allogeneic apoptotic cells.

**FIGURE 1:**
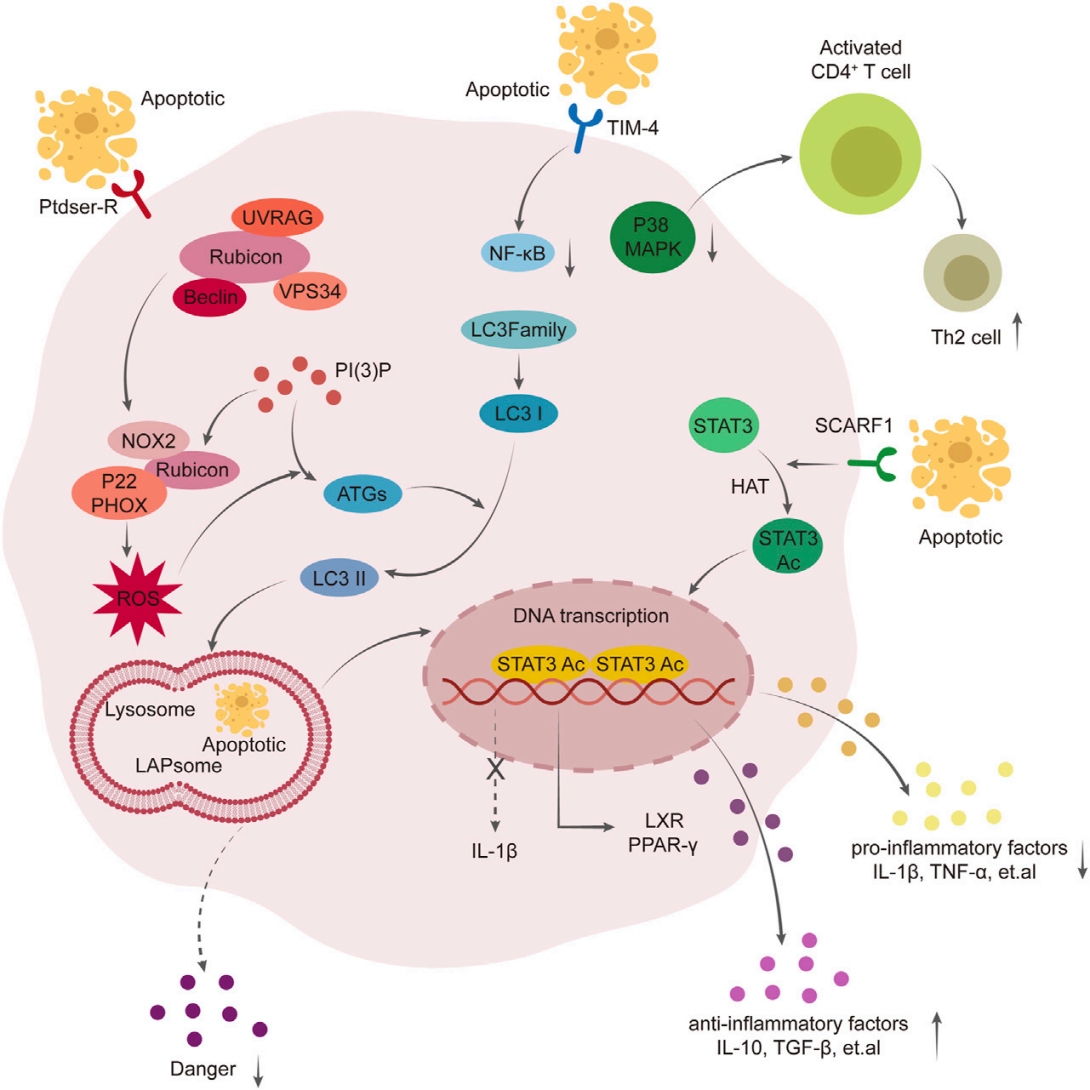
Mechanism of phagocytosis of Kupffer cells in the formation of immune tolerance. Overexpression of Rubicon mediates LAP pathway, promotes the production of ROS and translocation of LC3- Ⅱ, thus promotes the fusion of LAPsome and Lysosome, enhances the scavenging ability of Kupffer cells to apoptotic T cells, and further activates PPAR γ pathway to exert anti-inflammatory effect. Scavenger receptor class F member 1 (SCARF1) enhances the phagocytosis of Kupffer cells through calcium-dependent PI3K-AKT-STAT3 signal pathway mediated by acetylation of STAT3. Overexpression of Tim-4 mediates NF-kB, MAPK pathway to enhance the migration ability of macrophages and neutrophils, enhance the phagocytosis of apoptotic T cells, promote the proliferation of Th2, and improve liver injury after liver transplantation.

**FIGURE 2:**
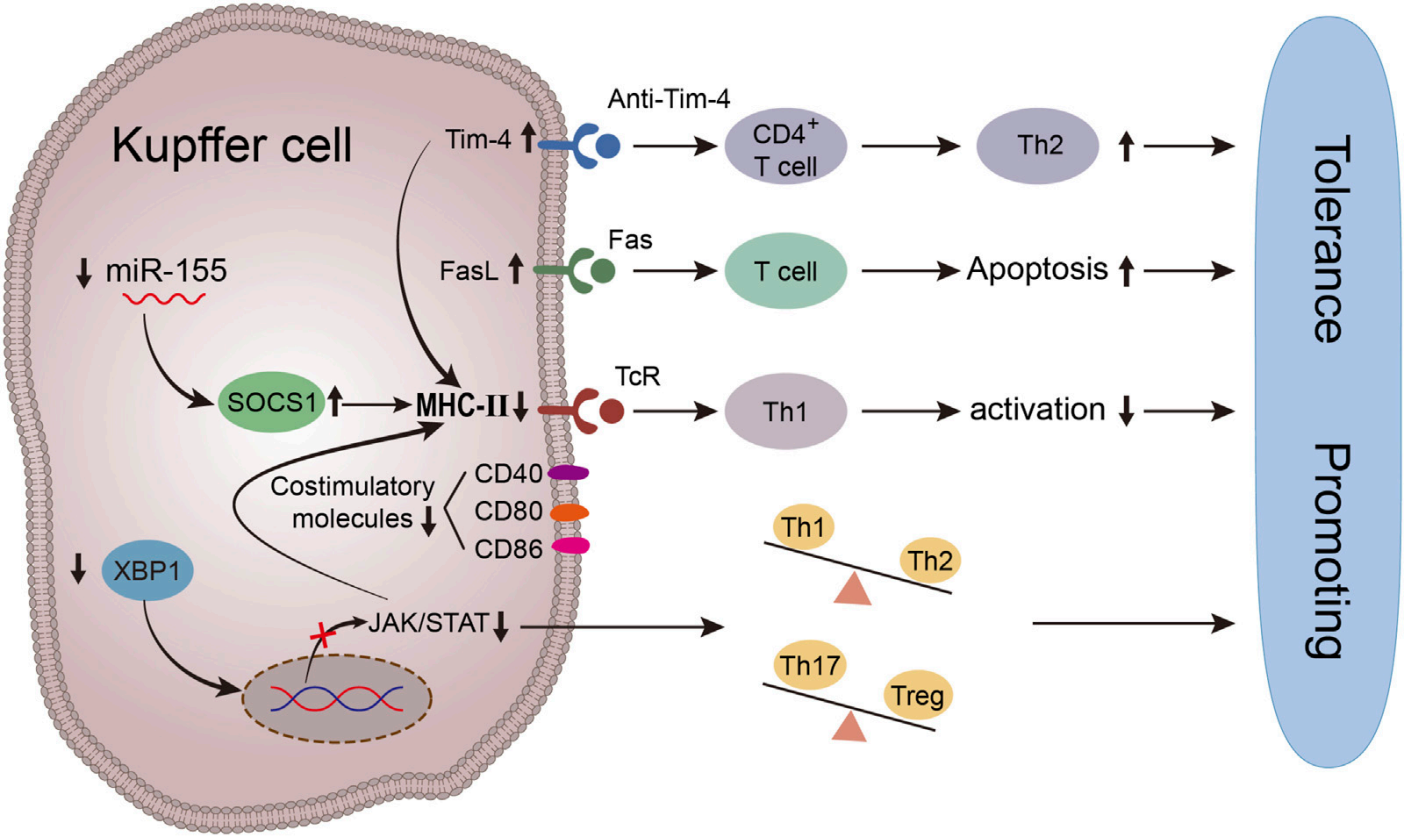
Schematic diagram of immune tolerance environment induced by antigen presentation function of Kupffer cells. Up-regulating the expression of Tim-4 in KCs *in vitro* can reduce the secretion of Th1 pro-inflammatory cytokines and inhibit the expression of MHC- Ⅱ and costimulatory molecules on KCs by inhibiting the activities of LPS/TLR4/NF-kB, LPS/MAPK and IRF3, at the same time, Tim-4 can induce T cell apoptosis and negatively regulate the process of immune response by down-regulating the antigen-presenting ability of Kupffer cells. miR-155. Inhibition of miR-155 can upregulate the expression of SOCS1, keep Kupffer cells in a stable and immature state, reduce the surface expression of costimulatory molecules and MHC- Ⅱ molecules, downregulate T cell response and pro-inflammatory cytokine secretion, and reduce the proliferation of allogeneic T cells, increase the expression of FasL, thus induce T cell apoptosis. Inhibition of XBP1 expression in activated Kupffer cells inhibited the phosphorylation level of JAK1/STAT1. Thereby down-regulating the expression of MHC-Ⅱ, inhibiting its antigen-presenting ability, and reducing acute rejection.

Our study demonstrated that overexpression of Rubicon, significantly increased the proteins and ROS required for LC3-associated phagocytosis (LAP), as well as the recruitment of LC3II and the fusion of phagosomes with lysosomes. This effectively enhanced the scavenging ability of Kupffer cells on apoptotic T cells (Chen et al., 2022). Overexpression of F1 scavenger receptor (SCARF1) in Figures 1, 2 Kupffer cells enhances phagocytosis via the calcium-dependent PI3K-AKT-STAT3 signaling pathway, effectively reducing acute rejection after liver transplantation, increasing clearance of apoptotic cells, improving liver function and promoting immune tolerance post-transplantation (Xu et al., 2021). Promoting the expression of Tim4 hinders the migration and function of Kupffer cells and neutrophils, impedes TLR2/4/9-dependent signal activation, ameliorates liver injury post-transplantation, reduces the release of inflammatory cytokines such as tumor necrosis factor-α, interferon-γ, CCL2 and CXCL2, thereby mitigating immune rejection after liver transplantation (Ji et al., 2014; Wu et al., 2018).

Additionally, research conducted by BRENNER et al has demonstrated that the release of three immune antigens - calreticulin, CRT, ERp57 and GP96 - occurs during cell death. The phagocytosis of dead cells by Kupffer cells aids in the elimination of these antigens and is therefore beneficial in preventing rejection during allogeneic liver transplantation (Brenner et al., 2013).

### 3.2 The polarization of Kupffer cells is involved in the formation of immune tolerance

Like other macrophages, Kupffer cells (KCs) exhibit remarkable plasticity and display distinct cellular phenotypes in response to exogenous or endogenous signals detected by Toll-like receptors (TLRs) within different microenvironments, namely, M1 and M2 KCs. M1 type is induced by lipopolysaccharide and interferon-γ, leading to the secretion of pro-inflammatory cytokines such as interleukin-1β and tumor necrosis factor-α, as well as cytotoxic factors including nitric oxide synthase and reactive oxygen species. In addition, M1-type Kupffer cells are capable of secreting CXCL9, CXCL10 and other chemokines to recruit a large number of Th1 cells and induce severe immune responses (Chen et al., 2008; Mosser and Edwards, 2008). The M2 phenotype is stimulated by the cytokines IL-4 and IL-13, which enhance the expression of anti-inflammatory mediators including IL-10, scavenger receptors, mannose receptors and tissue remodeling molecules like TGF-β (Mantovani et al., 2013). Studies have demonstrated that activated M2 Kupffer cells can enhance the expression of apoptosis signaling molecules, including FasL, PD-L1 and Galectin-9, thereby inducing apoptosis of effector T cells. Secondly, M2 Kupffer cells can also promote the differentiation of Th2 cells to inhibit acute rejection after liver transplantation in rats. Therefore, it is of great significance to change the polarity of KCs to M2 type after liver transplantation for alleviating acute rejection and inducing immune tolerance of liver transplantation.

In our previous study, we discovered that Rubicon-mediated LC3-associated phagocytosis (LAP) facilitates Kupffer cells in the clearance of apoptotic T lymphocytes and activation of PPAR γ through polyunsaturated fatty acid (PUFA), a degradation product of apoptotic cells. This process exerts an anti-inflammatory and M2-promoting effect, thereby reducing acute rejection after liver transplantation (Chen et al., 2022). Inhibition of XBP1s in LPS-activated Kupffer cells (KCs) can upregulate the expression of arginase-1 (Arg-1) and CD204, downregulate the expression of MHC-II and CD40, and induce a phenotypic shift towards an M2-like phenotype via activation of JAK3-STAT6 signaling pathway. Notably, upregulation of XBP1s triggers the expression of M1-associated cytokines via the JAK1-STAT1 signaling pathway (Zhang et al., 2021a). Neutrophil extracellular traps (NETs) are reticular structures formed by neutrophils. The level of serum NETs significantly increases after liver transplantation and is negatively correlated with liver function. Nets exacerbates acute rejection following liver transplantation by inhibiting DNase-1 and promoting M1 polarization of Kupffer cells, as well as inducing intracellular translocation of high-mobility group box-1 (HMGB1) (Liu et al., 2022). By upregulating the expression of F1 scavenger receptor in Kupffer cells, intracellular calcium efflux can be increased, PI3K-AKT-STAT3 signaling activity can be upregulated, and ultimately M2 polarization of Kupffer cells can be promoted. This leads to a reduction in pro-inflammatory factor secretion and an increase in anti-inflammatory factor secretion, which is beneficial for immune tolerance formation after liver transplantation (Xu et al., 2021). IL-34 can induce M2 polarization of Kupffer cells via the activation of PI3K/Akt/mTOR pathway signaling *in vivo* after liver transplantation, and it can alter the phenotype of Kupffer cells from M1 to M2 *in vitro*, thereby inhibiting acute immune rejection (Zhao et al., 2018). The expression of Soluble fibrinogen-like protein 2 (sFGL2) is upregulated in a rat orthotopic liver transplantation tolerance model and positively correlates with the frequency of M2 Kupffer cells. In the *in vivo* experiment of acute rejection model, adeno-associated virus-mediated expression of FGL2 (AAV-FGL2) effectively mitigates acute rejection (Pan et al., 2018). Recently, it has been discovered that miR-449a can directly target PLOD1 mRNA and inhibit the NF-κB signaling pathway, thereby suppressing M1 polarization of Kupffer cells (Cao et al., 2023). Additionally, miR-505-5p induces M2 polarization of macrophages through the MyD88/TRAF6 axis to mitigate acute rejection following liver transplantation (Chai et al., 2022).

After co-culturing Kupffer cells with bone marrow mesenchymal stem cells (MSCs), You et al. observed a significant reduction in the expression of pro-inflammatory cytokines MHC II, CD40, CD80, and CD86 in Kupffer cells. At the same time, there was an increase in the expression of anti-inflammatory cytokines, including transforming growth factor β, interleukin 4, prostaglandin 2 and interleukin 10. In a rat liver transplantation model with bone marrow mesenchymal stem cells present, there was observed a phenotype shift from M1 to M2 in Kupffer cells along with increased immune tolerance (Zhang et al., 2021c).

### 3.3 Antigen presentation function of Kupffer cells is involved in the formation of immune tolerance

Major histocompatibility complex (MHC) molecules are the primary antigens responsible for rejection following transplantation. Prolonged inflammatory stimulation post-liver transplantation can induce upregulation of MHC class I expression in all nucleated liver cells and stimulate MHC class II expression in antigen-presenting cells (APCs) (Ronca et al., 2020). Kupffer cells are a type of highly efficient antigen-presenting cell that can express both MHC class I molecules to present endogenous antigens to CD8^+^ cytotoxic T cells and MHC class II molecules to present exogenous antigens to CD4^+^ helper T cells (Thomson and Knolle, 2010). The T cell antigen receptor (TCR) specifically recognizes the antigen peptide-MHC molecular complex and binds CD4 to MHC-class II molecules, leading to down-activation of associated tyrosine kinase CD3 and CD4, which initiates a cascade reaction. Subsequently, T lymphocytes recognize and bind with multiple pairs of costimulatory molecules on the surface of APC. Following dual activation signals from MHC-II and costimulatory molecules, PLC-PKC, IP3, and PI3K-Ras-MAPK signaling pathways activate various transcription factors to promote the proliferation and differentiation of T lymphocytes (Sckisel et al., 2017). After activation, T lymphocytes can differentiate into various helper T lymphocytes, including Th1, Th2, Th17 and Tregs. Maintaining the balance between Th1/Th17 and Th2/Treg is fundamental to normal immune function; any aberration or imbalance in this process may lead to a range of immune disorders (Ni et al., 2017). Cytokines released by Th1, such as IL-2 and IFN-γ, and those released by Th17, such as IL-17 and IL-23, can promote the accumulation of inflammatory cells, increase the release of inflammation-related cytokines, accelerate hepatocyte necrosis and apoptosis, and positively participate in immune regulation. These cytokines are closely associated with ischemia-reperfusion injury and the development of acute rejection after transplantation and can impede Th2 differentiation. Conversely, cytokines released by Th2 cells such as IL-4, IL-10, and IL-13, as well as those released by Tregs including IL-10 and transforming growth factor-β (TGF-β), have been shown to mitigate immune responses (Doherty, 2016). Therefore, regulating the antigen presentation function of Kupffer cells can facilitate immune tolerance and promote the release of chronic anti-inflammatory transmitters (Ellias et al., 2021).

Some studies have demonstrated that JAK-activated STAT1 and tyrosine phosphorylation can directly bind to the DNA binding site in the promoter of MHC-II gene, thereby enhancing its transcriptional activity (Li and Watowich, 2014). Our previous study revealed that X-box binding protein 1 (XBP1s) activation in Kupffer cells by LPS can augment the phosphorylation levels of JAK1 and STAT1, thereby upregulating MHC-II expression, indicating an enhanced antigen presentation ability of Kupffer cells. The inhibition of XBP1s in donor Kupffer cells may disrupt the Th paradigm balance by inducing apoptosis of Th1 and Th17 cells post-transplantation, thereby promoting immune tolerance and preserving allogeneic liver function (Zhang et al., 2021a).

TIM-4 is the sole non-T cell TIM protein that exists in antigen presenting cells. Certain studies have demonstrated that liver transplantation can enhance the expression of TIM-4 in activated Kupffer cells. Silencing TIM-4 expression in Kupffer cells impedes phosphorylation of p65 and p38 in the nuclear factor kappa B and p38MAPK signaling pathways, thereby significantly ameliorating acute rejection injury *in vivo*. Moreover, TIM-4 in Kupffer cells attenuates IL-4 expression and impedes the STAT6/Gata3 signaling pathway to disrupt transcription of human forkhead box protein P3 (Foxp3) and elevate TGF-β levels, while promoting iTregs conversion (Wu et al., 2018).

As the target gene of miR-155, Suppressor of cytokine signaling-1 (SOCS1) serves as a central regulator in immune cell function and inflammatory response. It plays a crucial role in regulating the activation, development, and differentiation of Kupffer cells and dendritic cells (Alexander, 2002). After miR-155-mediated silencing of SOCS1, a significant increase in the expression of MHC-II and costimulatory molecules on Kupffer cells’ surface was observed, thereby enhancing their antigen presentation function. It was also discovered that miR-155 regulates the expression level of SOCS1 in Kupffer cells. In contrast, upregulation of SOCS1 in conjunction with miR-155 inhibition maintains Kupffer cells in a stable and immature state, resulting in decreased surface expression of costimulatory and MHC-II molecules. This leads to reduced reactivity of antigen-specific T cells and secretion of proinflammatory cytokines, as well as diminished proliferation of allogeneic T cells. Additionally, it induces T cell apoptosis and promotes the development of immune tolerance (Li et al., 2014).

### 3.4 Surface molecules of Kupffer cells participate in the formation of immune tolerance

#### 3.4.1 The role of apoptosis factor Fas-FasL (Fas/Fasligand) in immune tolerance of liver transplantation

Fas and FasL are a pair of transmembrane proteins that trigger apoptosis. Upon specific binding of FasL to Fas, apoptotic signals are transmitted to target cells, leading to the demise of T cells expressing Fas. Activated KCs via the NF-κB pathway not only induce cytokine and immune adhesion molecule production for immune regulation, but also promote FasL expression, thereby inducing T lymphocyte apoptosis. The findings indicated a significant upregulation of FasL expression in acute rejection, accompanied by an increase in cytotoxic T lymphocyte activity. Notably, the highest number of apoptotic hepatocytes and bile duct cells was observed on postoperative day 7 in the rejection group. In addition, a significant number of infiltrating T cells underwent apoptosis. Following the blockade of KCs function with gadolinium trichloride, the level of FasL decreased and T lymphocyte apoptosis was reduced. These findings suggest that Fas/FasL interaction-induced apoptosis is a crucial mechanism underlying liver injury caused by rejection after transplantation (Martinez and Krams, 1999; Pan et al., 1999).

Miyagawa-Hayashino have confirmed the high expression of FasL in Kupffer cells, thus providing further evidence to support this finding (Miyagawa-Hayashino et al., 2007). The number of apoptotic fragments and FasL-positive Kupffer cells in the portal vein was significantly higher in the acute rejection group compared to both the chronic rejection and inflammatory control groups. The apoptotic fragments phagocytized by FasL (+) Kupffer cells were predominantly CD4 (+) interferon gamma (+) T cells, while the population of FasL (+) Kupffer cells decreased upon administration of immunosuppressants or steroids. Kupffer cells can also suppress the activity of T cells and decrease the survival rate of Th1-like T cells that produce interferon-gamma, resulting in immunosuppression within the portal system. This may drive an immune response towards a Th2 phenotype in allogeneic liver transplantation, thereby affecting the establishment of immune tolerance. Additionally, it has been observed that the expression of FasL on Kupffer cells is positively correlated with the RAI score of acute rejection, indicating a significant increase in FasL expression as the severity of acute rejection worsens. Therefore, phagocytosis of apoptotic T cells by FasL (+) Kupffer cells can serve as an index for evaluating human liver allograft rejection activity.

SUN infused Kupffer cells from long-term surviving rats after allogeneic liver transplantation into acute rejection model rats and observed that they could induce alloantigen specificity, thereby prolonging the survival time of the allograft. Kupffer cells can induce Th1 apoptosis by upregulating FasL, modulate cytokines produced by Kupffer cells or T cells, and enhance the secretion of anti-inflammatory cytokines such as interleukin-10 and transforming growth factor β to promote a shift towards Th2/Th3 response (Sun et al., 2003).

We observed a significant increase in both FasL mRNA and protein levels within Kupffer cells of the transplantation group compared to the control group. Furthermore, we found that activation of NF-κ B and expression of FasL were positively correlated with IL-4 production, confirming that overexpression of FasL may enhance immune response following liver transplantation (Chen et al., 2008). In brief, NF-κB can upregulate the expression of FasL and IL-4 in Kupffer cells, trigger T cell apoptosis via the Fas/FasL pathway, and modulate helper T cell subset differentiation to facilitate immune tolerance induction following liver transplantation.

The aforementioned studies indicate that the Fas/FasL pathway plays a crucial role in establishing immune tolerance following liver transplantation. On the one hand, FasL-positive Kupffer cells phagocytose a large number of inflammatory cytokines and downregulate T cell activity during acute rejection after transplantation. This drives the immune response towards an inhibitory phenotype, promoting the establishment of immune tolerance. Whether the expression of FasL in Kupffer cells can serve as a reliable indicator for human liver allograft rejection requires extensive clinical data. Conversely, nuclear factor kappa B and Fas-670AA genotypes may impact Tc apoptosis post-transplantation, thereby influencing immune tolerance formation; however, the underlying mechanism remains unclear and warrants further investigation.

#### 3.4.2 The role of Toll-like receptors in immune tolerance of liver transplantation

TLRs are transmembrane protein family members that function as pathogen recognition receptors, detecting both endogenous and exogenous molecules. They play a crucial role in coordinating the innate immune response and subsequent adaptive immune response. The liver’s normal physiological environment is characterized by innate immune tolerance. The human body transports TLR2 and TLR4 ligands from the intestinal tract to the liver parenchyma via the portal circulation, thereby maintaining immune homeostasis through downregulation of TLR expression and signal transduction on Kupffer cells’ surfaces. In liver transplantation rejection, the subgroup of TLRs consisting of membrane-bound pattern recognition receptors (PRRs) can recognize pathogen-associated molecular patterns (PAMPs) or damage associated molecular patterns (DAMPs). This recognition leads to the release of TLR stimulating signals that promote dendritic cell maturation and antigen presentation. Additionally, this process plays a crucial role in recruiting and activating T and B cells, thereby linking the innate and adaptive immune systems. These processes are essential for transplant tolerance and rejection (Vidya et al., 2018; Timmons et al., 2020).

Stimulation of pathogen-associated molecular patterns (PAMPs) on Kupffer cells via Toll-like receptors (TLR2, 3, 4 and 9) can elicit the production of a diverse array of cytokines, including tumor necrosis factor-α, interleukin-1 β, interleukin-6, interleukin-12, and interleukin-10. This in turn triggers the secretion of transforming growth factor-β, platelet-derived growth factor (PDGF), matrix metalloproteinases and reactive oxygen species to modulate immune tolerance establishment post-transplantation. The signal cascade is initiated by four key cohesive molecules: myeloid differentiation protein-88 (MyD88), Toll interleukin receptor domain-containing adaptor protein-inducing interferon β (TRIF), Toll interleukin receptor-associated protein (TIRAP) and Toll interleukin receptor domain-containing adaptor protein-inducing IFN-β-related (TRAM). The TIR domain is linked with one of these to induce an immune response (Mencin et al., 2009).

Peng et al. demonstrated a significant increase in TLR4/MD-2 mRNA and protein expression, as well as tumor necrosis factor α levels, 2 hours post-transplantation. However, treatment with anti-TLR4 antibodies resulted in a significant reduction of these factors, suggesting a potential relationship between the antibody and TLR4 inhibition. It is associated with the inhibition of TLR4/MD-2 gene and protein expression in Kupffer cells, subsequently interfering with TNF-α production (Peng et al., 2004). KESHAVARZ et al. observed an increase in the expression levels of TLR2 and TLR4 7 days after liver transplantation, indicating acute rejection occurrence (Keshavarz et al., 2021). Additionally, Oetting et al. conducted an analysis on the TLR4 gene in 738 cases of allogeneic liver transplant recipients from various ethnic backgrounds and concluded that donor polymorphism in TLR4 may serve as a crucial factor regulating TLR4 activity, thereby impacting the risk of graft loss and immune rejection (Oetting et al., 2012). De La Fuente et al. investigated the pathology of transplanted livers in 159 patients with hepatocellular carcinoma who underwent liver transplantation, and identified an association between the TLR91486TT genotype and a reduced risk of liver cancer recurrence post-transplantation. Therefore, the TLR91486C/T polymorphism may serve as a potential biomarker for identifying patients with low risk of HCC recurrence prior to liver transplantation (de la Fuente et al., 2019).

The aforementioned studies have demonstrated that Toll-like receptors (TLRs) modulate the production of diverse cytokines and orchestrate immune rejection post-transplantation by engaging adapter molecules. However, further refinement is required to enhance the reliability of predicting acute rejection based on TLR2 and TLR4 expression levels after transplantation. Many studies have confirmed that TLR4 expression and donor polymorphism can serve as indicators of immune rejection after transplantation. However, further clinical data and research verification are required to determine the relationship between the TLR9-1486TT genotype and liver cancer recurrence post-transplantation in Table 1.

**TABLE 1:** The role of surface molecules of Kupffer cells in immune tolerance of liver transplantation.

Surface molecules	Author/Year	Impact on LT	Possible mechanism
Fas/FasL	Martinez and Krams (1999), Pan et al. (1999), Chen et al. (2008)	Overexpression of FasL can enhance immune response after liver transplantation	NF-κB can upregulate the expression of FasL and IL-4 in Kupffer cells, trigger T cell apoptosis via the Fas/FasL pathway, and modulate helper T cell subset differentiation to facilitate immune tolerance induction following liver transplantation
	Miyagawa-Hayashina et al. (2007)	The expression of FasL on Kupffer cells is positively correlated with the RAI score of acute rejection, indicating a significant increase in FasL expression as the severity of acute rejection worsens	Downregulate the activity of T cells, reduce the survival rate of Th1-like T cells producing interferon-γ, and drive the immune response in allogeneic liver to develop to Th2 (inhibitory) phenotype
	Sun et al. (2003)	To prolong the survival time of liver transplantation by producing alloantigen specificity	Upregulate FasL to induce apoptosis in Th1 and regulate the secretion of cytokines from Kupffer cells or T cells, such as interleukin-10 and transforming growth factor-β, and transform them into Th2/Th3
Toll-like receptors	Peng et al. (2004)	The expression of TLR4/MD-2 mRNA and protein and the level of tumor necrosis factor-α increased significantly at 2 h after transplantation	Anti-TLR4 antibody inhibits the expression of TLR4/MD-2 gene and protein in Kupffer cells, which interferes with the production of tumor necrosis factor-α
	Keshavarz et al. (2021)	TLR2 and TLR4 can be used as biomarkers to predict the occurrence of acute rejection after liver transplantation	Uncertain
	Oetting et al. (2012)	Donor polymorphisms in TLR4 are important factors regulating TLR4 activity, which may affect the risk of graft loss and the occurrence of immune rejection	Uncertain
	de la Fuente et al. (2019)	TLR9-1486C/T polymorphism may help to identify patients with low risk of liver cancer recurrence before liver transplantation	Uncertain

### 3.5 Discussion

#### 3.5.1 Contributions and limitations of current research on the mechanism of Kupffer cells in immune tolerance

The establishment of immune tolerance following liver transplantation represents the optimal outcome for transplant recipients, yet its intricate connections and underlying mechanisms remain incompletely elucidated. The majority of current strategies for inducing immune tolerance focus on T cells and involve the use of immunosuppressive agents. However, prolonged administration of these drugs not only imposes a financial burden on patients but also elicits numerous adverse effects that compromise their post-transplant immune function. Long-term exposure to low levels of a substance can lead to complications such as infection, tumor formation, and drug toxicity. How to effectively utilize immunosuppressants for inducing postoperative immune tolerance remains uncertain. Kupffer cells, due to their unique anatomical location and physiological function, play an indispensable role in regulating the immune response after liver transplantation. Due to its unique anatomical location and physiological function, Kupffer cells play an indispensable role in immune regulation not only within the liver but also throughout the entire body. Their functional mechanisms, including cell phagocytosis, polarization, antigen presentation, and membrane proteins are crucial for establishing immune tolerance after transplantation. After being stimulated by various factors, Kupffer cells play a dual role in transplant rejection: they act as direct antigen presenters and release numerous pro-inflammatory factors to activate T cells, exacerbating inflammation; meanwhile, they also induce T cell apoptosis and promote T cell proliferation and differentiation in diverse directions. Additionally, Kupffer cells in their M2 polarization state can secrete various immunosuppressive factors to collectively facilitate the establishment of transplantation tolerance. However, despite ongoing experimental research on Kupffer cells, translating these findings into clinical practice remains a challenging direction that requires further effort.

#### 3.5.2 The novelty and limitation of the review

Currently, numerous reviews have discussed Kupffer cells; however, the majority of literature is limited to their phenotype conversion, function and role in other diseases. The mechanisms of Kupffer cells in liver transplantation are primarily based on experimental methods. Many of the mechanisms remain unclear, and as such, clinical applications cannot be drawn. Additionally, varying methods of literature retrieval may result in incomplete collections or an overabundance of outdated sources lacking innovation. This review is based on a plethora of original studies conducted by our research group. While presenting recent advancements in Kupffer cell research, it also delves into the mechanisms behind their role in inhibiting rejection and inducing/maintaining immune tolerance during liver transplantation. A comprehensive overview of how different functions of Kupffer cells contribute to the formation of immune tolerance will provide valuable insights for future research.
